# A multiport DC-to-DC converter-driven inductive wireless charging system for EVs with integrated photovoltaic and energy storage systems

**DOI:** 10.1038/s41598-025-07420-9

**Published:** 2025-07-03

**Authors:** Aganti Mahesh, Bharatiraja Chokkalingam, C. Santhakumar, K. Sathiyasekar, Sanjeevikumar Padmanaban

**Affiliations:** 1 Centre for Electric Mobility (CEM), Department of Electrical and Electronics Engineering, SRM Institute of Science and Technology, Kattankulathur, Chengalpattu, Tamil Nadu 603203 India; 2 Depertment of Electrical and Electronics Engineering, K S R COLLEGE OF ENGINEERING, KSR Kalvi Nagar, Tiruchengode, Tamil Nadu 637215 India; 3 Depertment of Electrical Engineering, Information Technology and Cybernetics, University of Southern-Eastern Norway, Porsgrunn, Norway

**Keywords:** Wireless power transfer, Half-bridge converter, Dynamic wireless charging, Electric vehicle and resonant converter, Electrical and electronic engineering, Batteries

## Abstract

This paper introduces an innovative three-port DC–DC converter (TPC)-based wireless charging system (WCS) that seamlessly integrates photovoltaic (PV) and an energy storage system (ESS). The proposed system leverages the advantages of an isolated topology, enhancing safety, reducing electromagnetic interference, and enabling flexible power management. The regulation of input ports from PV and ESS (battery) is achieved through a pulse width modulation switching scheme, ensuring stable voltage across the WCS port. The isolated design also enables bidirectional power flow at the ESS port under specified conditions, facilitated by auxiliary switches. The WCS port incorporates series–series and LCC-S compensation, ensuring efficient power transfer under various misalignment and load conditions. The proposed system is validated through simulation using MATLAB and Ansys Maxwell, demonstrating its dynamic performance and reliability. Additionally, experimental results confirm the operational modes of the TPC topology and evaluate the behavior of the integrated PV and wireless battery system. This study highlights the advantages of an isolated power architecture, offering a robust and efficient solution for standalone applications.

## Introduction

Wireless Power Transfer (WPT) has emerged as a transformative solution to overcome the limitations associated with Electric Vehicles (EVs) charging. It enables on-the-go charging, effectively reducing the need for large, costly batteries with extended charging times. This innovative wireless charging system (WCS) provides fully autonomous and continuous charging capabilities^1^.

In recent years, the adoption of isolated photovoltaic (PV) systems for distributed power generation has grown significantly. This trend is largely driven by the increasing demand for sustainable energy solutions, particularly in remote locations such as electric vehicle (EV) charging stations. However, due to the intermittent nature of solar energy, integrating an energy storage system (ESS) has become essential to ensure a reliable power supply. Among various ESS options, batteries have gained widespread use in stand-alone PV systems, playing a crucial role in balancing the harvested solar energy with the charging demands, especially in off-grid locations^2^.

Non-isolated converters are widely used in power conversion applications due to their compact design and high efficiency. However, they also pose several challenges. One of the primary issues is the shared grounding between ports, which can lead to ground loops and electromagnetic interference (EMI), negatively impacting system performance. Additionally, the absence of galvanic isolation restricts their use in applications where electrical separation is necessary for safety and noise reduction. Managing power flow between multiple ports while ensuring stability and efficiency presents another challenge, requiring sophisticated control mechanisms. Cross-coupling effects between ports can introduce unwanted interactions, potentially leading to system instability and necessitating advanced control algorithms. Furthermore, non-isolated converters have a limited voltage conversion range compared to their isolated counterparts, making them less suitable for high step-up or step-down applications. Addressing these challenges requires innovative converter designs and advanced control strategies to improve performance, reliability, and safety, particularly in isolated photovoltaic (PV) systems^3,4^.

To accommodate the varying voltage levels of PV modules, batteries, and chargers, engineers typically employ either two independent DC–DC converters or an integrated three-port converter (TPC). The TPC offers several advantages over independent converters, including a more compact structure, reduced component count, and lower overall weight. TPC configurations can be classified into three main types based on their level of isolation: non-isolated^5^, partially isolated (hybrid coupling)^6,7^, and fully isolated^8^. Non-isolated TPCs operate without a transformer, offering a simpler design with high efficiency but lacking electrical isolation. Partially isolated TPCs incorporate a two-port transformer, balancing efficiency with some level of electrical separation. Fully isolated TPCs, on the other hand, use a three-port transformer, ensuring complete electrical isolation between ports, and making them suitable for applications that demand enhanced safety and noise immunity.

The isolated three-port converter (TPC) has garnered considerable attention for DC fast-charging applications. In certain implementations, dual-transformer-based topologies are employed to achieve significant improvements in soft-switching performance compared to conventional configurations^9^. However, these designs tend to increase system cost and operational complexity. Other variations, such as half-bridge and multilevel converter-based TPCs, have also been explored. While multilevel TPCs can offer enhanced performance, they further increase design complexity and control challenges^10,11^. Recent studies have concentrated on using resonant LLC-type soft-switching approaches to increase the performance and efficiency of isolated multiport converters in response to the growing need for high power density^12,13^.

Integrating a high-frequency transformer within the TPC not only ensures isolation between ports but also facilitates voltage step-up or step-down for the high-frequency AC output generated by converters interfacing with low-voltage PV and battery ports. While several three-port converter topologies have been developed for integrating renewable energy sources such as PV, fuel cells, and wind turbines with energy storage systems, there is limited research on their application in wireless charging systems. Further studies are needed to explore and optimize three-port converters for wireless power transfer, enhancing their efficiency and applicability in modern energy systems.

A three-port converter designed to integrate an ESS and a WPT system is proposed in^14^. This approach utilizes a hybrid coupling method, where two half-bridge converters connect the photovoltaic (PV) and ESS ports to supply the WPT system. A two-port high-frequency transformer provides isolation to two of the ports, ensuring safe and efficient energy transfer. Alternatively, a three-port DC–DC converter approach integrates two DC energy sources with a DC load port using a three-winding high-frequency transformer. This method can be extended by incorporating an inverter at the DC load port, providing isolation across all three ports, a key advantage over hybrid coupling. Several studies^15–17^ have explored three-winding high-frequency transformer-based three-port systems integrated with an ESS for plug-in charging systems. However, when the input voltages across these three ports do not match ideally, circulating power and current increase, potentially compromising soft-switching capabilities under varying input voltage and load conditions.

Misalignment remains a critical challenge in WPT systems, affecting power transfer efficiency and system stability. Various compensation techniques have been proposed to mitigate these issues and enhance performance. Double-coupler circuits or transformers are often employed in WPT systems to improve energy transfer reliability, particularly in dynamic charging applications^18–20^. In^21^, an intermediary coupler circuit was introduced, allowing independent control of individual charging sections and enabling frequency synchronization without introducing unwanted reactive power onto the backbone supply. Similarly^22^, explored the use of three matching transformers to connect the cascaded inverter output to the transmitter coil, enabling precise current control.

Selecting an appropriate compensation topology significantly impacts both the efficiency of a PV-integrated three-port conversion system and its ability to handle load variations and misalignment conditions. Series–series (SS) and LCC-S (Inductor-Capacitor-Capacitor-Series) compensation are among the most widely used techniques^23^. Both methods employ the same secondary-side compensation topology, but their primary-side resonance compensation strategies result in distinct system characteristics. A crucial distinction emerges when operating near the zero-phase angle (ZPA) frequency—SS compensation exhibits constant current source characteristics on the secondary side, whereas LCC-S compensation maintains a constant voltage source behavior^3,24,25^. However, misalignment introduces variations in coupling coefficient and transferred power, affecting SS and LCC-S compensation differently. In SS compensation, misalignment results in reduced current transfer, directly impacting the load. In contrast, LCC-S compensation, with its voltage-source characteristics, experiences fluctuations in voltage output under misalignment conditions, potentially affecting the system’s ability to maintain stable voltage regulation. Understanding these differences is crucial for optimizing WPT systems, as the choice between SS and LCC-S compensation depends on whether current stability or voltage regulation is the primary design requirement.

This paper introduces a TPC TPC-based integrated Photovoltaic (PV) system, that incorporates wireless charging capabilities and an energy storage system. The study includes an analysis of the impact of series–series (SS) and LCC-S compensation. The effectiveness of the proposed system is validated through simulations conducted using Ansys Maxwell and MATLAB-Simulink.

The key contributions of this research are as follows:

- Development of a three-port converter (TPC) for inductive wireless power transfer (WPT) systems, enabling efficient multi-port energy management.
- Integration of photovoltaic (PV) and energy storage systems (ESS) to ensure continuous charging, enhanced power utilization, and improved system stability under varying input conditions.
- Comparative analysis of series–series (SS) and Inductor-Capacitor-Capacitor-Series (LCC-S) compensation techniques, highlighting their distinct impacts on system efficiency, power transfer characteristics, and misalignment tolerance in the proposed integrated TPC system.

The structure of the paper is organized as follows: Section “Proposed three-port DC–DC converter (TPC) topology” introduces the TPC designed to integrate PV and a battery to power an autonomous wireless charging system. Sections “Operational analysis of the proposed system” present a detailed analysis of the operation modes during battery charging and discharging, respectively. Simulation and Experimental results and analysis are done in Section “Simulation and experimental analysis”, the paper concludes with a summary in Section “Conclusion”.

## Proposed three-port DC–DC converter (TPC) topology

**Fig. 1:**
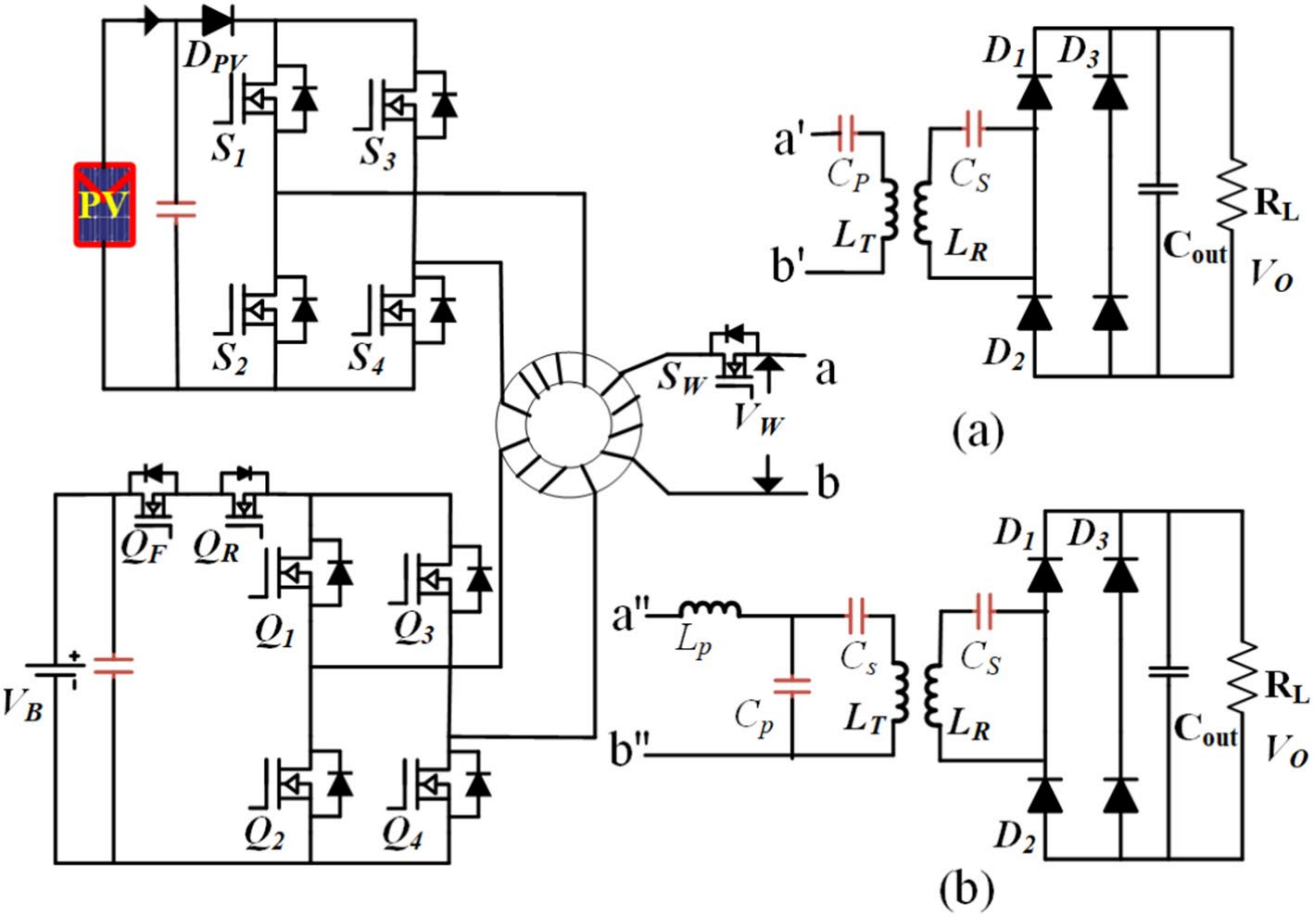
Model representation of the proposed three-port DC–DC converter (TPC) wireless charging system (WCS) system. (**a**) SS compensation. (**b**) LCC-S compensation.

In Fig. 1, we observe the proposed Photovoltaic (PV) interfacing system, which centers on an isolated three-port DC/DC converter. This innovative converter is built around a high-frequency transformer, serving as the focal point of the system’s architecture. Within this system, the PV module and the battery pack are each linked to distinct windings of the high-frequency transformer, facilitated by individual H-bridge inverters working with 85 kHz frequency as per the SAE J2954. Furthermore, a separate pole is connected to the transmitter of the wireless charging system and is equipped with a switch (S_W_). The number of turns in the windings for each port PV, battery pack, and transmitter are represented as NPV, NB, and No, respectively. To ensure smooth operation, a diode (D_PV_) is integrated into the PV module’s configuration. This diode plays a crucial role by preventing any circulating current from flowing back into the battery port. Its presence guarantees unidirectional power flow, ensuring that power generated by the PV module is directed solely towards the intended destination. In contrast, the battery pack is connected to an H-bridge through a bidirectional switch (Q_R_, Q_F_). This switch serves multiple functions, including the management of zero circulating current when the PV port converter is operational during discharging. Additionally, it facilitates control over the charging process, offering flexibility and efficiency in the system’s overall operation.

In WCS, as stated transmitting coil is connected to switch S_w_, as depicted in the figure. To reduce the leakage current through the transmitting coil and excite the system when the receiver coil is there. Disconnected when misalignment is more than desired. S_w_ connected transmitter coil through series compensation.

In this configuration, the WCS is equipped with series–series (SS) and LCC-S compensations. In the case of SS compensation, the primary side switch (S_W_) is connected to compensation components C_P_, and the secondary side compensation is denoted as C_S_. For LCC-S compensation, the primary side switch S_W_ is connected to components L_p_, C_p_, and C_s_, while the secondary side is connected to component C_s_. On the receiver side of the system, a high-frequency diode rectifier is employed. As depicted in Fig. 1 for SS compensation terminals ab connected to a′b′ and for LCC-S terminals ab connected to a′′b′′.

As shown in Fig. 1, the control objectives of the proposed system revolve around two primary aspects: tracking the maximum power point of the PV module and maintaining precise regulation of the output charging voltage while preventing any circulating current between the PV and battery ports. The PV port operates under the governance of Maximum Power Point Tracking (MPPT), tasked with extracting the maximum available power from the PV module and directing it towards the transmitting coil. The Perturb and Observe (P&O) based MPPT technique is employed for this purpose, adjusting the duty cycle of the PV port inverter, denoted as d_PV_. In the PV port H-bridge inverter, switches S3 and S4 are complemented each half-cycle, while the pulse width of switches S1 and S2 is modulated according to d_PV_ to control the power flow from the PV port. Concurrently, the battery port is responsible for regulating the transmitter voltage and power to the desired level, regardless of the operational status of the PV module. This task is accomplished through the use of a straightforward Proportional-Integral (PI) controller. The PI controller governs the load voltage, v_O_, by effectively managing the duty cycle of the energy storage system (ESS) port inverter, d_B_. Importantly, this approach ensures that the battery port is adaptable and capable of either supplementing the deficiency in harvested PV power or absorbing the surplus power generated by the PV module. This adaptability is crucial, especially in the face of variable weather conditions and load fluctuations.

The ESS enters the discharging mode of operation when the PV power is less than the required power for the WCS. The identical PWM switching scheme employed for the PV inverter is applied to the ESS port inverter, but with a duty cycle denoted as d_B_. Simultaneously, the forward switch S_F_ is activated to facilitate power transfer from the battery port to the WCS port, ensuring that the reverse switch Q_R_ is deactivated. This prevents the formation of a path for circulating current that may flow from the PV port to the ESS in the scenario where V_PV_ < V_B_.

During the charging operation of the (ESS), the H-bridge of the ESS port is deactivated, and the forward switch Q_F_ is turned off, while the reverse switch Q_R_ is modulated using d_B_ to permit reverse power flow. Consequently, the ESS port functions as a buck converter fed from a body-diodes rectifier. Noteworthy that d_B_ is measured from the falling edge of the modulating signals of the PV port, S_1_ and S_2_, during which the PV port is enabled to both charge the battery and feed the wireless charging port. Furthermore, the gating signal for Q_R_ is extended to the end of the half-cycle period to facilitate the discharge of stored energy in the leakage inductance of the winding into the battery. The bidirectional power flow operation of the battery port is controlled by the sign of d_B_. The Proportional-Integral (PI) controller is configured to generate positive values for d_B_ during the discharging operation, facilitating forward power flow, and negative values for reverse power flow during the charging operation.

Summing up the above processes, the operation of the proposed system can be categorized into three modes based on the status of the PV port and the battery: In the first mode, the PV system simultaneously supplies power to both the WCS port (load) and the ESS/Battery. In this scenario where PV alone supplies power to the WCS port is rare, as PV power typically aligns with the load power only for a brief period during the total daily operation. The second mode initiates when the power from the PV port is less than that required by the WCS port. In this mode, both the PV port and the ESS port contribute power to the WCS port. The third mode involves the ESS port exclusively supplying power to the WCS port.

## Operational analysis of the proposed system

There are different stages in each mode of operation for the proposed TPC-based WCS. Neglecting the windings resistances, the magnetizing current, and the voltage drop across switches, the converter model, referred to as the winding of the PV port, can be written as:

1 $${L}_{PV}d{i}_{PV1}/dt={v}_{PV1}-E$$

2 $${L}_{b}^{\prime}d{l}_{b1}^{\prime}/dt={v}_{b1}^{\prime}-E$$

3 $${L}_{w}^{\prime}d{i}_{w}^{\prime}/dt=E- {v}_{w}^{\prime}$$

4 $${i}_{w}^{\prime}={i}_{PV1}+{i}_{b1}^{\prime}$$

where the suffix ′ indicates referring to the winding of the PV port, *L*_PV_, $L_{B}^{\prime} = \left( {N_{PV} /N_{B} } \right)^{2} L_{B}$ ,$L_{w}^{\prime } = \left( {N_{PV} /N_{o} } \right)^{2} L_{w}$ and $L_{T}^{\prime } = \left( {N_{PV} /N_{t} } \right)$ are the leakage inductances of the transformer. Winding voltage and currents of PV indicated by $v_{pv1,}$ $i_{pv1}$.For battery $v_{b1}^{\prime } = \left( {N_{PV} /N_{t} } \right)v_{b1}$_._,$i_{b1}^{\prime } = \left( {N_{PV} /N_{t} } \right)i_{b1}$ and WCS system

$$v_{w1}^{\prime } = \left( {N_{PV} /N_{t} } \right)v_{w1} ,\quad i_{w1}^{\prime } = \left( {N_{PV} /N_{t} } \right)i_{w1}$$

The voltage across WCS port V_W_ can be derived following way.

By considering V_O_ as the output voltage or EV battery voltage and P_O_ as the output power of the charging system or WCS power. The battery can be presented as DC resistance (R_L_) as:

5 $${R}_{L}=\frac{{{V}_{o}}^{2}}{{P}_{o}}$$

Receiver coil Self-inductance can be calculated with the help of secondary coils quality factor (Q_s_)

6 $${Q}_{s}=\frac{{w}_{o}{L}_{R}}{{R}_{A}}$$

where w_0_ resonant frequency and R_a_ (the equivalent AC resistance) at the input side of the rectifier are presented as

7 $${R}_{A}=\frac{8}{{\pi }^{2}}{R}_{o}$$

Similarly, rms current flowing through the secondary coil is given as

8 $${I}_{srms}=\frac{{V}_{srms}}{{R}_{L}}$$

The mutual inductance of magnetic coupler ‘M’ can be calculated as

9 $${w}_{o}M{I}_{Wp}={{R}_{L}L}_{R}$$

Rms current flowing through primary coil I_prms_ can be calculated using

10 $${I}_{wrms}=\frac{{P}_{o}}{{V}_{Wrms}}$$

*V*_*Wrms*_ presents the voltage across the WCS port.

Compensation capacitances *C*_*P*_*, C*_*S*_ can be calculated by using the following expression of resonant frequency that is given by

11 $${w}_{o}=\frac{1}{{L}_{T}{C}_{P}}=\frac{1}{{L}_{R}{C}_{S}}$$

For the same inductance values, LCC-S compensation can be calculated from the following equations,

12 $${V}_{srms}=\left|j{w}_{0}M{I}_{w}\right|=\left|j{w}_{0}M\frac{\raisebox{1ex}{$2$}\!\left/ \!\raisebox{-1ex}{$\pi {V}_{in}$}\right.}{j{w}_{0}{L}_{p}}\right|=\left|\frac{2k\sqrt{{L}_{T}{L}_{R}}}{\pi {L}_{p}}\right|$$

Primary side series filter inductance can be calculated from the following formula.

13 $${L}_{pa}=\sqrt{\frac{{k}_{max}{V}_{prms}{U}_{srms}}{{w}_{0}{P}_{max}}{L}_{p}}$$

The parallel capacitor r from the primary side calculated from,

14 $${L}_{p}{C}_{p}=\frac{1}{{{w}_{0}}^{2}}$$

Series compensation of the primary side given as,

15 $${L}_{T}-{L}_{p}=\frac{1}{{{w}_{0}}^{2}{C}_{s}}$$

**Fig. 2:**
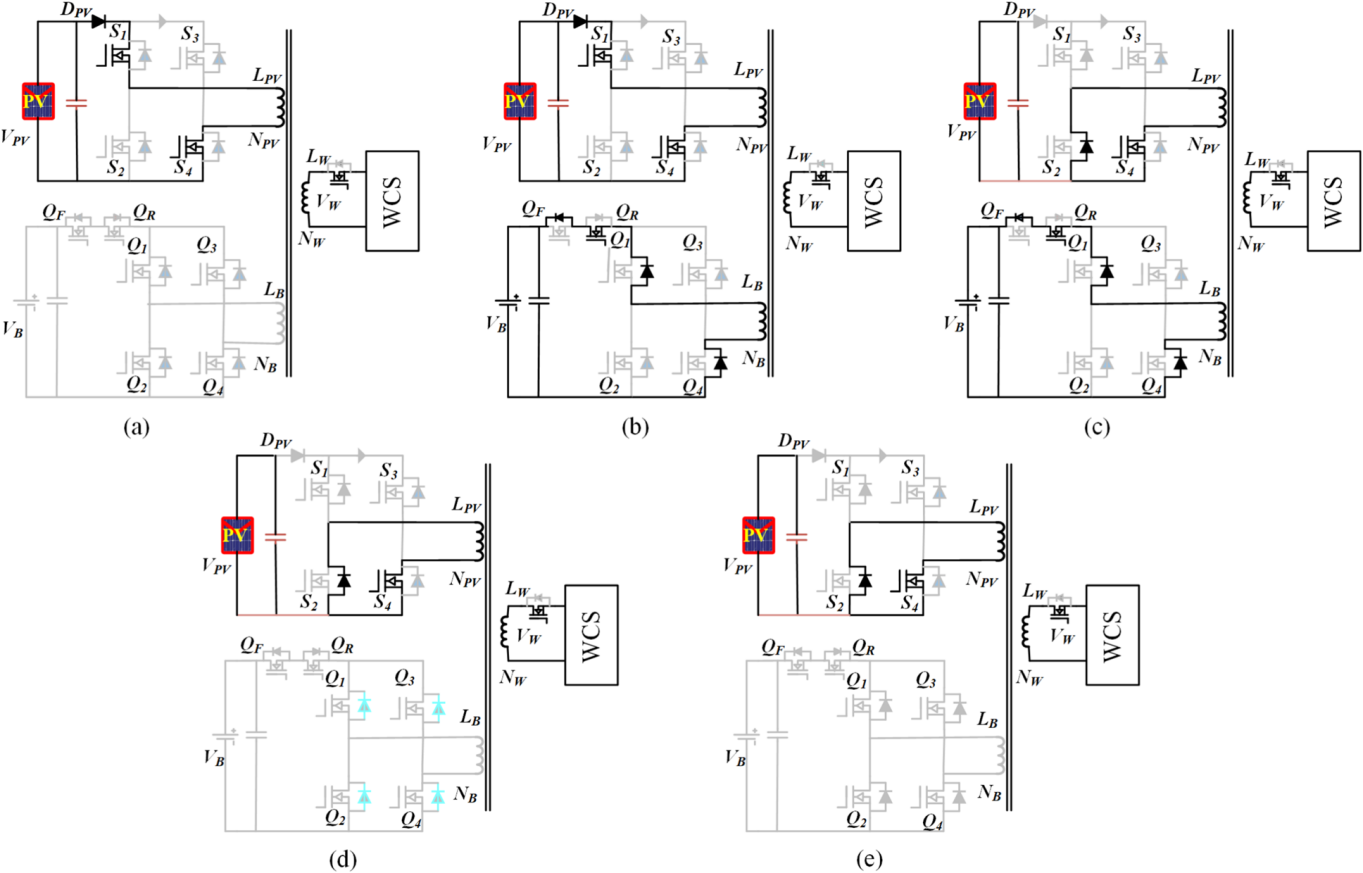
Illustration of the sub-states in Mode-1 through circuit switching in the model.

**Fig. 3:**
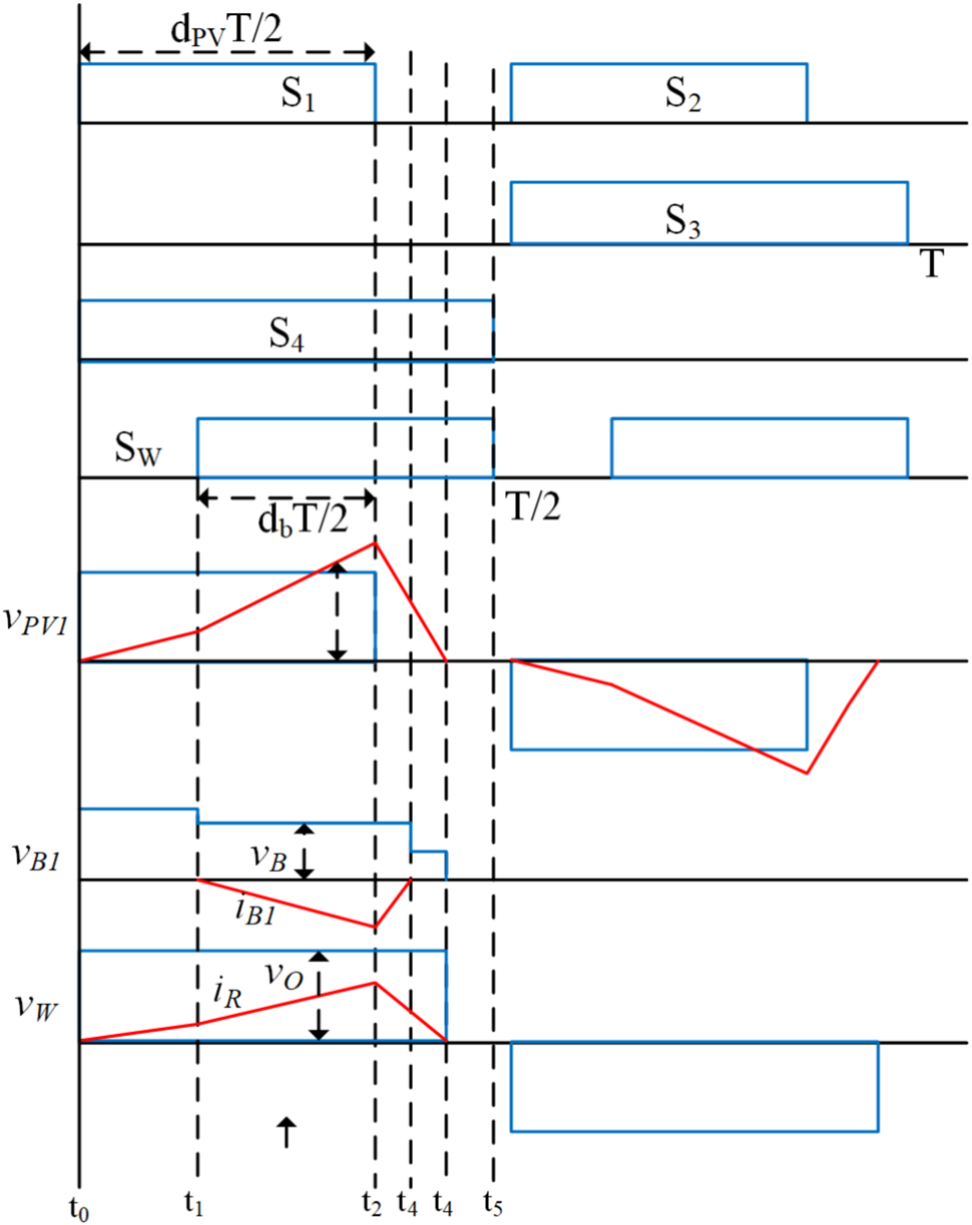
Representation of the sub-states in Mode-1 with voltage and current waveforms of the model.

In the first mode, various sub-states of the system during ESS charging are illustrated per positive half-cycle in Fig. 2. The corresponding current and voltage waveforms of the transformer windings are depicted in Fig. 3.

**Fig. 4:**
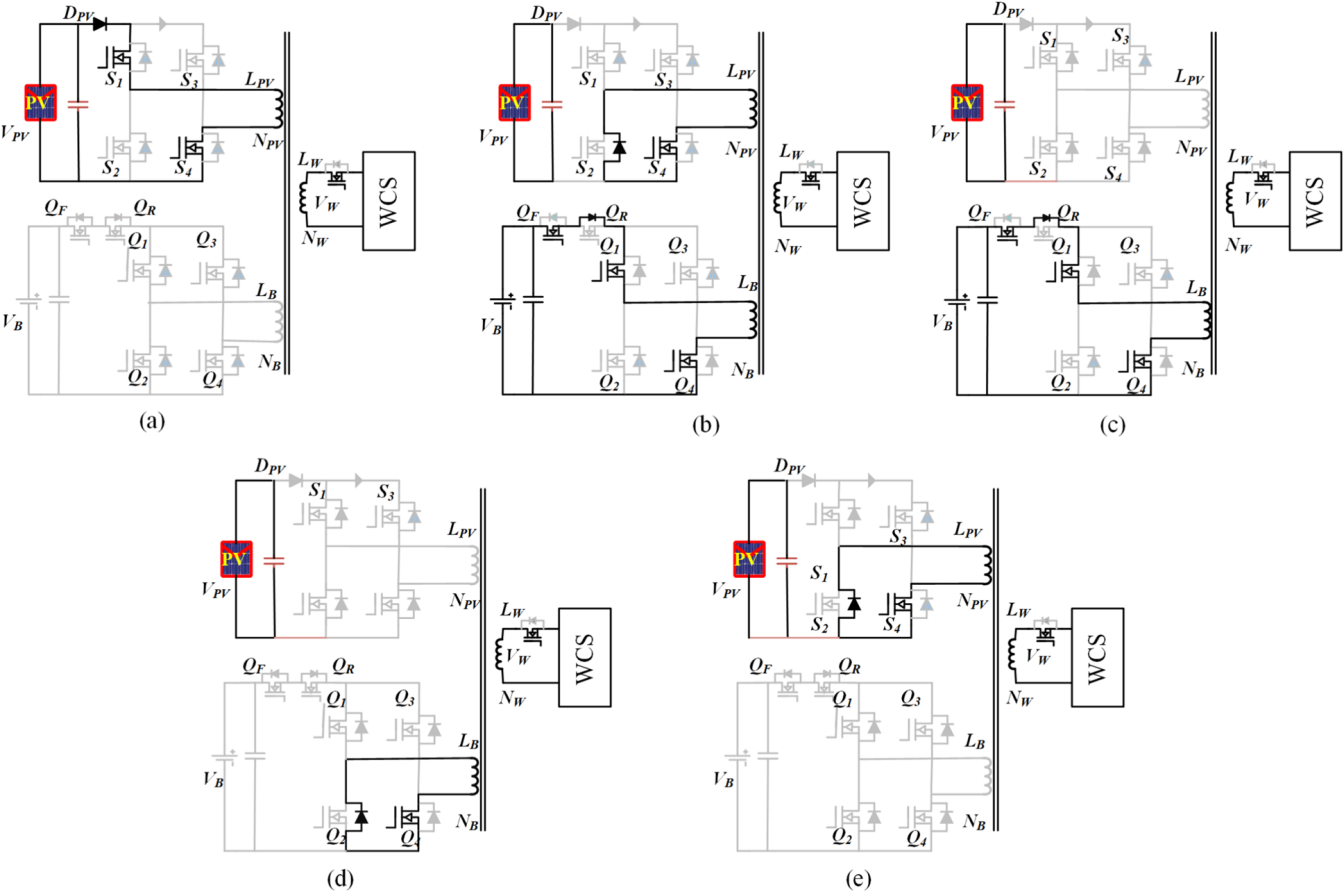
Illustration of the sub-states in Mode-2 through circuit switching in the model.

**Sub-state 1 (t**_**0**_** ≤ t < t**_**1**_**)**: In this state, switches S_1_ and S_4_ of the PV port inverter are turned on, while the ESS port switches are in the off condition. During this state, the generated PV power is transferred to the WCS port. Additionally, the switch related to the WCS port, denoted as S_W_ is turned on to power the transmitter coil of the WCS, as illustrated in the Fig. 4. The corresponding rising current is presented in the Figure. By substituting i′_b1_ = 0 and v′_w_ = v′_o_ from Eqs. (1), (3), and (4), the PV current at the end of the mode can be expressed as

16 $${I}_{PV1}=\frac{\propto ({d}_{PV}-{d}_{B})}{2f{L}_{PV}} ({v}_{PV}-{v}_{o}^{\prime})$$

where, α = L_PV_/L_PV_ + L′_W_ , V_PV_ is the PV module voltage, and V′_W_ is the voltage across V_W_ referred to as the PV port.

**Sub-state 2 (t1 ≤ t < t2)**: This process is initiated when PV power is more than the WCS power. Q_R_ is turned on at t_1_ to direct the excessive power PV from ESS, Fig. 2b. During the mode, the battery and PV ports’ current reaches a maximum at t_2_ which can be seen in Fig. 3. The battery and PV ports’ currents, I_B_ and I_PV_, are calculated by solving (1) to (4) and using (16) as:

17 $${I}_{B}=\frac{{d}_{B}{N}_{PV}}{2f({L}_{B}^{\prime}+\propto {L}_{W}^{\prime}){N}_{B}} [({1-\propto )v}_{PV}+{\propto v}_{o}^{\prime}-{v}_{B}^{\prime}]$$

18 $${I}_{PV2}=\frac{\propto ({d}_{PV}-{d}_{B})}{2f{L}_{PV}}\left\{{d}_{PV}\left({v}_{PV}-{v}_{o}^{\prime}\right)+\frac{{d}_{B}}{{\propto +L}_{B}^{\prime}/{L}_{W}^{\prime}}\left[({1-\propto )v}_{PV}+{\propto v}_{o}^{\prime}-{v}_{B}^{\prime}\right]\right\}$$

The initiation of the charging process is prompted by the condition E > v′_B_, giving rise to the following inequality,

19 $${N}_{PV}>\frac{{V}_{B}}{\left(({1-\propto )V}_{PV}/{N}_{PV}+{\propto V}_{o}/{N}_{o}\right)}$$

where V_B_ represents the maximum battery voltage, V_PV_ denotes the PV module voltage at the maximum power point, and V_o_ is the nominal load voltage. The turn ratio between the windings of the PV and load ports is chosen based on their respective voltage levels. Furthermore, the number of turns of the battery pack port winding (N_B_) is designed according to Eq. (19) to ensure the proper functioning of the charging operation.

**Sub-state 3 (t2 ≤ t < t3)**: at time t2, the modulating leg of the PV port inverter is turned off, as shown in Fig. 2c. The stored energy in the transformer charges the output port and battery. This mode continues till stored energy is completely discharged. The discharging current can be written as by (17) and (18) submitting in the (1) to (4)

20 $${I}_{PV3}=\frac{\propto }{2f{L}_{PV}}\left[{d}_{PV}\left({v}_{PV}-{v}_{o}^{\prime}\right)+{d}_{B}\left({v}_{o}^{\prime}-\frac{({1-\propto )v}_{PV}{v}_{o}^{\prime}}{{v}_{B}^{\prime}-{\propto v}_{o}^{\prime}}\right)\right]$$

**Sub-state 4 (t3 ≤ t < t4)**: In this phase, the battery port becomes floating, and the stored energy in the L_PV_ and L_W_ continues to supply power to the WCS port, as depicted in Fig. 2d.

**Sub-state 4 (t4 ≤ t < t5)**: In this stage, as shown in Fig. 2e, the high-frequency transformer is de-energized, with voltages and currents in the windings being negligible. The conclusion of this mode marks the initiation of the negative half-cycle by turning off S_4_ and S_R_, and turning on S_2_ and S_3_.

The power obtained from the PV module and the power delivered to the battery can be approximated by determining the average currents of the PV module and the battery pack, respectively, from Fig. 3. This estimation involves applying Eqs. (16), (17), and (18), yielding the following:

21 $${P}_{PV}=\frac{\propto {v}_{PV}}{4f{L}_{PV}}\left\{{d}_{PV}^{2}\left({v}_{PV}-{v}_{o}^{\prime}\right)+\frac{{d}_{B}^{2}}{{\propto +L}_{B}^{\prime}/{L}_{r}^{\prime}}\left[({1-\propto )v}_{PV}+{\propto v}_{o}^{\prime}-{v}_{B}^{\prime}\right]\right\}$$

22 $${P}_{B}=\frac{\left(1-\propto \right){d}_{B}^{2}}{4f{L}_{PV}({L}_{B}^{\prime}+\propto {L}_{r}^{\prime})}\left(\frac{({1-\propto )v}_{PV}}{{v}_{B}^{\prime}-{\propto v}_{o}^{\prime}}-1\right){v}_{PV}{v}_{o}^{\prime}$$

### Operation modes during battery discharging

**Fig. 5:**
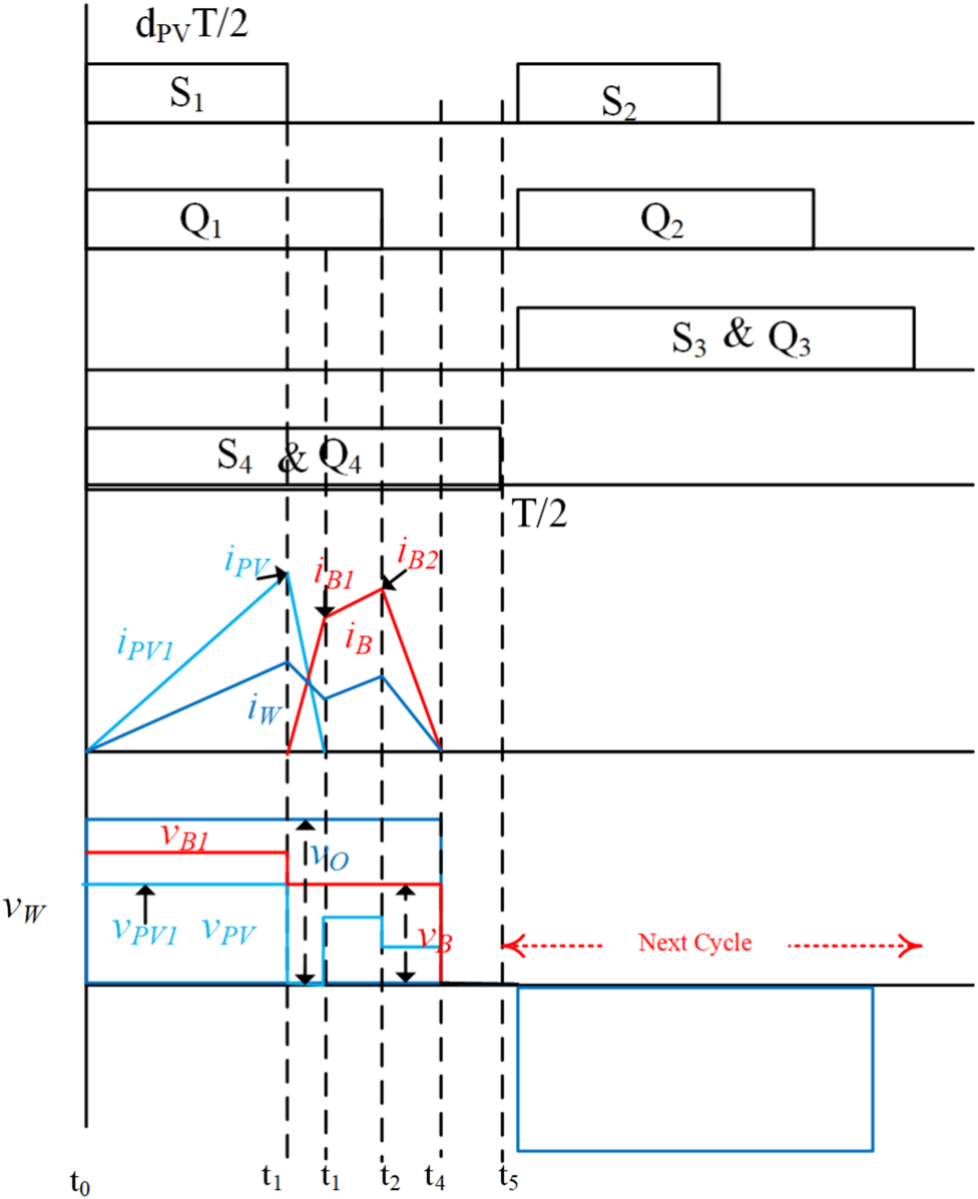
Representation of the sub-states in Mode-2 with voltage and current waveforms of the model.

In the event of shading, the load (WCS port) power is shared between the ESS port and the PV port. The proposed converter exhibits five operational modes per half-cycle during ESS discharging, as illustrated in Fig. 4. The current and voltage waveforms of the windings in the three ports are depicted in Fig. 5. The analysis of the discharge operation of the proposed.

TPC resembles that of a topology introduced by the authors, which relies on two-quadrant inverters.

**Sub-state 1 (t**_**0**_** ≤ t < t**_**1**_**)**: When the PV port generates less power than the required power for the WCS in shading conditions, both the PV and ESS ports commence supplying power to the WCS port. Specifically, S1, S4, Q1, and Q4 are all active, as illustrated in Fig. 4a. Additionally, the battery port switch SF and the WCS port switch SW are turned on. The condition E > V_B_, derived from Eq. (8), is established to initiate the charging process. During this stage, the current from the PV port continues to rise until t1, as depicted in the figure. The peak value of the current can be derived as follows:

23 $${I}_{PV}=\frac{\propto {d}_{PV}}{2f{L}_{PV}}\left({v}_{PV}-{v}_{o}^{\prime}\right)$$

**Sub-state 2 (t**_**1**_** ≤ t < t**_**2**_**)**: At t1, when the PV port inverter transitions to the zero state, the stored energy in the inductor is released to charge the WCS port. Concurrently, the ESS port current increases to supply the load side. The current at the battery port at the end of this stage is expressed as follows:

24 $${I}_{PV1}=\frac{\propto {d}_{PV}{N}_{PV}}{2f{L}_{PV}{N}_{B}}\left({v}_{PV}-{v}_{o}^{\prime}\right)\frac{\left({L}_{B}^{\prime}+{L}_{r}^{\prime}\right){v}_{B}^{\prime}-{L}_{PV}{v}_{o}^{\prime}}{{L}_{B}^{\prime}{v}_{o}^{\prime}+{L}_{r}^{\prime}{v}_{b}^{\prime}}$$

**Sub-state 3 (t**_**2**_** ≤ t < t**_**3**_**)**: During this stage, the ESS continues to feed the WCS port side until the ESS port is switched to the zero state, as demonstrated in Fig. 4c. Since the PV port is floating, v_PV1_ = E which is less than v1b due to the voltage drop across the leakage inductance L_b_ and the turns ratio N_pv_/N_b_ which is less than unity. Resolving (2), (3), and (4) and substituting in (24), the peak current of the battery port is given by:

25 $${I}_{B2}=\frac{{N}_{PV}}{2f\left({L}_{B}^{\prime}+{L}_{W}^{\prime}\right){N}_{B}}\left\{{L}_{PV}\left[\left(1-\propto \right){v}_{PV}+\propto {v}_{o}^{\prime}-{v}_{b}^{\prime}\right]+{d}_{b}\left({v}_{b}^{\prime}-{v}_{o}^{\prime}\right)\right\}$$

**Sub-state 4 (t**_**3**_** ≤ t < t**_**4**_**)**: During this stage, the ESS port inverter is switched to the zero state, facilitating the transfer of stored energy in L_B_ to the WCS port through S_4b_, the body diode of S_2b_, and the output rectifier, as shown in Fig. 4d. By solving Eqs. (2) to (4) and incorporating (25), the process can be expressed as follows.

**Sub-state 4 (t**_**4**_** ≤ t < t**_**5**_**)**: The high-frequency transformer becomes de-energized, similar to Mode 5 of battery charging operation in Fig. 4e. Throughout the negative half-cycle, which commences after this mode, there exist five modes of operation. These modes are analogous to the positive half-cycle modes, nevertheless with distinct operating switches.

As presented in Fig. 1. The harvested power from the PV module and the power fed from the battery pack are given by:

26 $${P}_{PV}=\frac{\propto {d}_{PV}^{2}}{4f{L}_{PV}}{v}_{PV}({v}_{PV}-{v}_{o}^{\prime})$$

27 $${P}_{B}={d}_{B}\left[\frac{1}{2}{({d}_{B}-d}_{PV})\left({\text{I}}_{B1}-{\text{I}}_{B2}\right)-{\text{I}}_{B2}.f{t}_{PV,d}\right]$$

## Simulation and experimental analysis

A laboratory prototype of the TPC-based WCS has been implemented, with specifications and parameters for the TPC-based high-frequency transformer detailed in Table 1. A 600W maximum power PV prototype was developed using the Itech 6018B 500120 emulator, while the same emulator was utilized for the ESS system’s battery emulation. The system parameters for the 350W WCS are documented in Table 2. The HFI on the PV port side uses four SiC power MOSFETs (SCT2080KE) as active switches (S_1_–S_4_). The ESS port-side converter also employs SCT2080KE MOSFETs to facilitate charging. On the secondary side of the WCS, a high-frequency rectifier made up of four OnSemi-RURG30120 is used to convert AC to DC, powering the battery’s equivalent resistance.

**Table 1:** Comparison multi-coil magnetic couplers.

Description	PV port	ESS Port	WCS
Voltage level	54	54	48
Nominal Power	500	500	350
Leakage inductance	9 µH	6.5 µH	6 µH
Transformer winding turns	8	16	11
Switching frequency	85 kHz

**Table 2:** Comparison multi-coil magnetic couplers.

Parameters	Values
Power	350 W
Frequency	85 kHz
V_o_	48 V
V_inDC_	60 V
I_prms_	6.5 A
I_srms_	8 A
R_L_	5.3 Ω
R_B_	6.6 Ω
L_T_	63.4 µH
L_R_	40 µH
k	0.248
Series compensation	
C_P_	55 nF
C_S_	88 nF
LCC-S compensation	
L_p_	14 µH
C_p_	250 nF
C_s_	71 nF
C_S_	88 nF

**Fig. 6:**
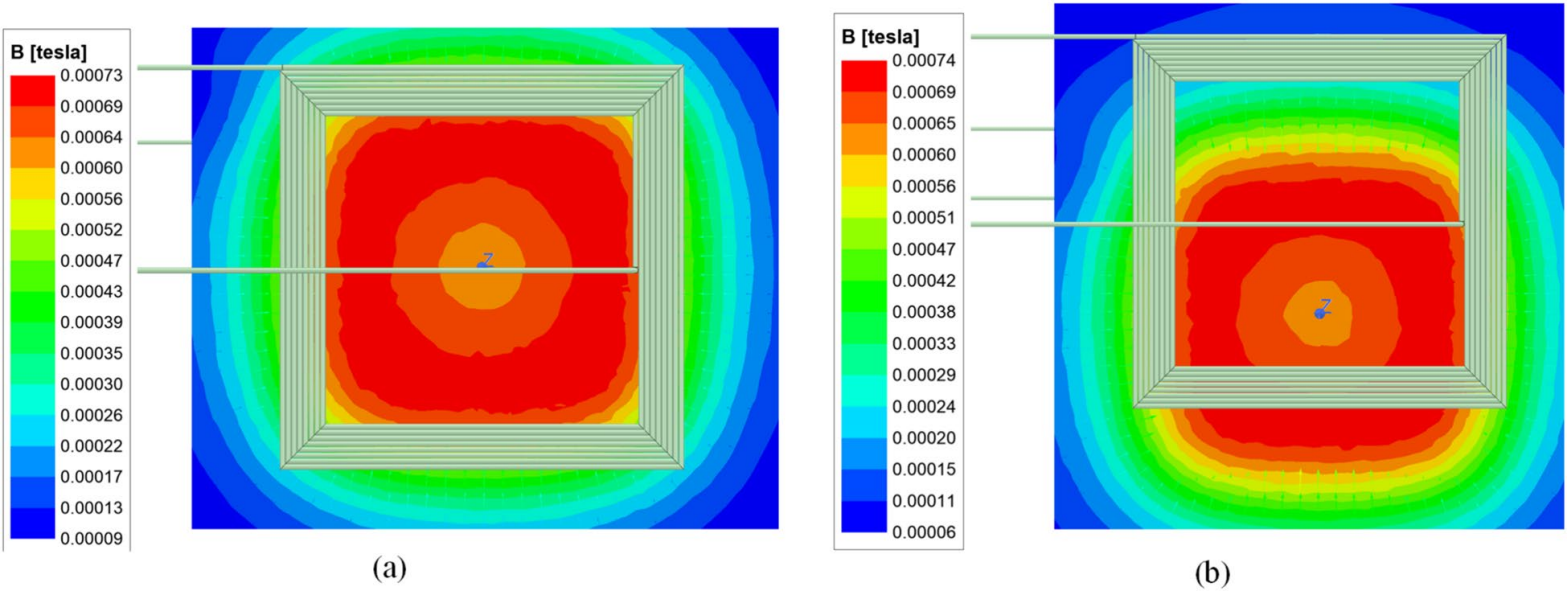
Ansys flux density distribution (**a**) Full-alignment (**b**) At 25% misalignment.

**Fig. 7:**
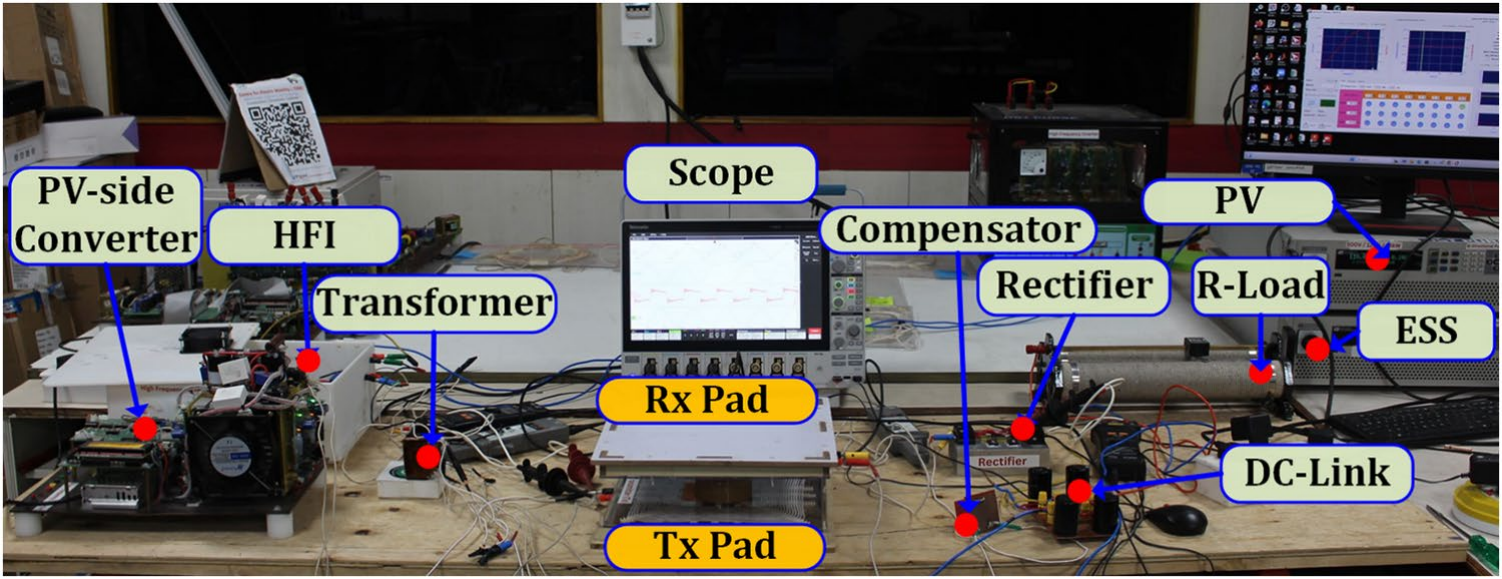
Experimental setup of proposed TPC system.

A square coil was selected for the design of the WCS. The mathematical calculations were verified using Ansys Maxwell simulation. The coils were designed based on the parameters tabulated in Table 2, and the FEA flux simulation models of the selected square pads are presented in Fig. 6. The number of turns required, based on both the mathematical analysis and FEM analysis, were predicted and wound accordingly. A comparison of the turns obtained from mathematical, FEA models, and practical systems is provided in Table 3. Figure 7 presents the experimental setup of the proposed system.

**Table 3:** No turns of WPT pads.

Parameters	Values
Coil length/width	18 cm
Conductor width: transmitter	0.25 cm/300 strands
Conductor width: Receiver	0.35 cm/620 strands
Turn gap	0.01 cm
Gauge of the single-strand	38AWG/0.101 mm
No. of Turns transmitter	13
No. of Turns Receiver	09

**Fig. 8:**
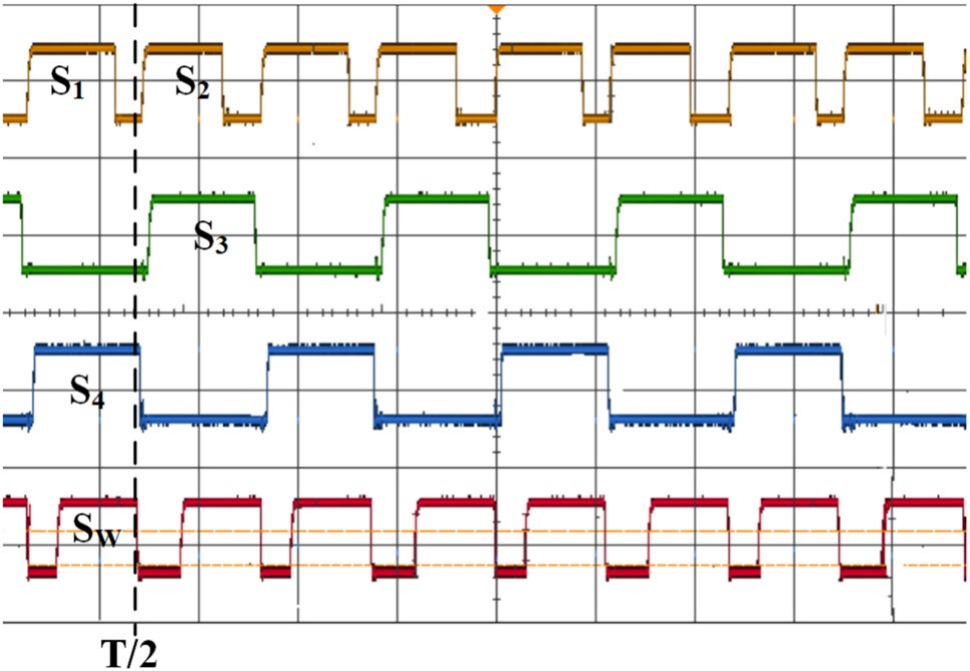
Representation of the switching states in Mode-1 Operation.

**Fig. 9:**
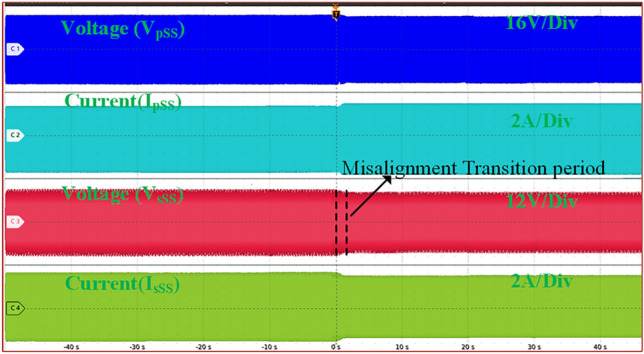
SS compensated the system’s AC output waveforms.

Figure 8 presents the switching pattern of the proposed topology when the ESS is charged by the PV-based high-frequency converter. Figure 9 illustrates the input and output waveforms for the WCS magnetic coupler using SS compensation, which presents full alignment to a 15% misalignment transition. The top first two waveforms present input waveforms and the next two present output waveforms. As the system moves into misalignment, the primary current increases with SS compensation, while the other waveforms show a decline.

**Fig. 10:**
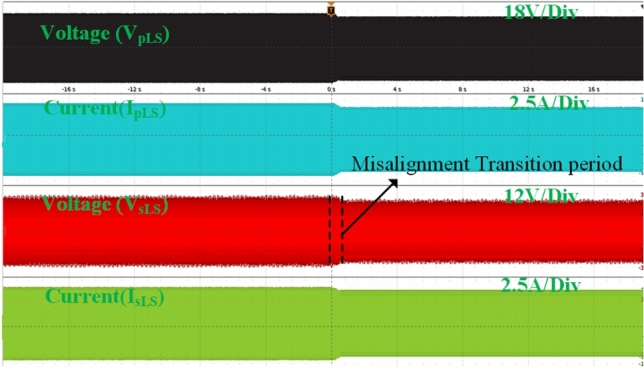
. LCC-S (LS) compensated system’s AC output waveforms.

**Fig. 11:**
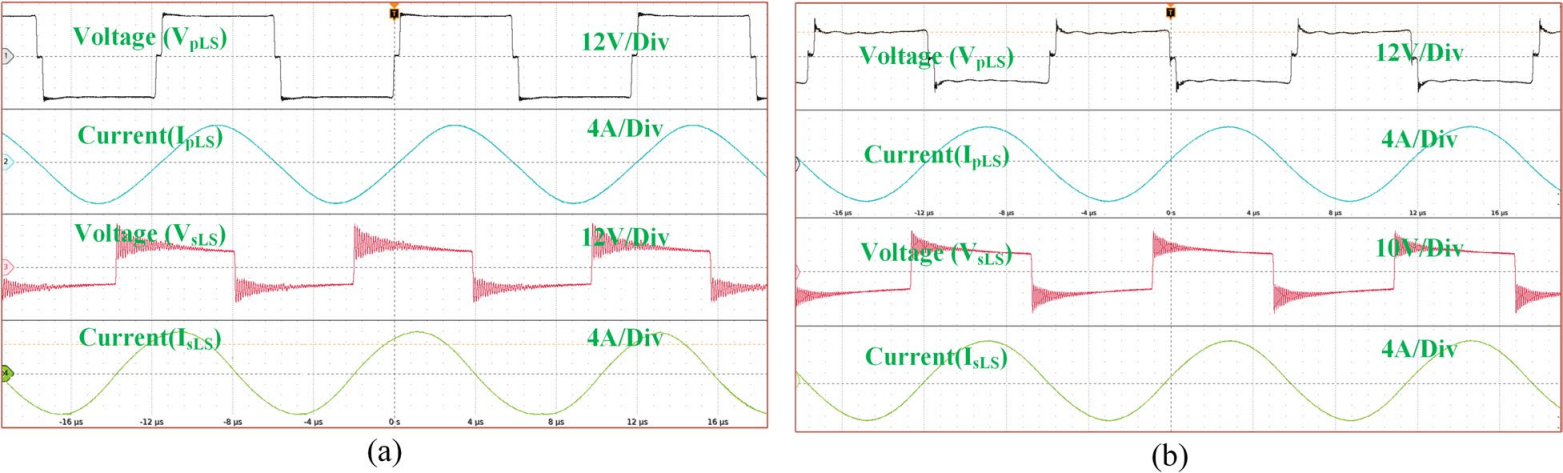
. LCC-S (LS) compensated the system’s AC output waveforms (**a**) At 10% misalignment and (**b**) At 25% misalignment.

Figure 10 presents the same waveforms for the magnetic coupler with LCC-S compensation, where a drop in all waveforms occurs after misalignment. Comparing the results, SS compensation demands more power under identical misalignment conditions. Figure 11 illustrates the AC output waveforms of the LCC-S (LS) compensated system under misalignment conditions. In Fig. 11a, at 10% misalignment, the waveform remains in the ZPA condition, ensuring efficient power transfer. However, in Fig. 11b, at 25% misalignment, the ZPA condition is slightly misplaced, indicating a minor phase shift and potential efficiency reduction.

**Fig. 12:**
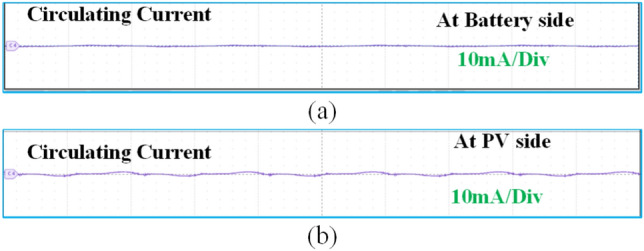
Circulating currents (**a**) At Battery side (**b**) PV side.

**Fig. 13:**
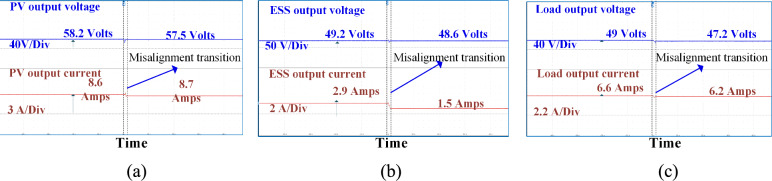
SS compensated the system’s DC output waveforms at (**a**) At the PV port input of the inverter (**b**) At the ESS port (**c**) At load resistance.

**Fig. 14:**
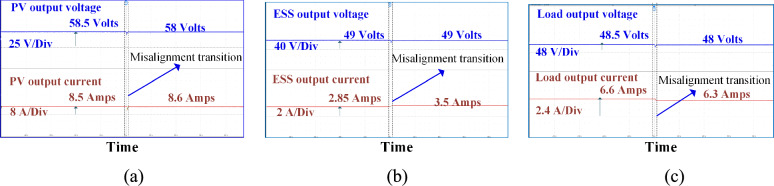
LCC-S compensated the system’s DC output waveforms at (**a**) the PV port input of the inverter (**b**) At the ESS port (**c**) At load resistance.

Figure 12 illustrates the circulating currents measured across the battery and PV ports when they are not in operation. As observed, the currents passing through these ports are negligible, measured only in milliamps (mA). Figure 13 presents the DC output waveforms of the proposed system with SS compensated system at misalignment condition. Figure 13a presents the output voltage and current waveforms at PV fed to the high-frequency inverter. In misalignment conditions, PV output voltage and efficiency system decreases. Figure 13b, c present the voltage and current waveforms at the ESS port and load side of the WCS port respectively. After the misalignment condition of the voltage and current waveforms, Fig. 14 presents the DC output waveforms of the proposed system with SS compensated system at misalignment condition. Figure 14a presents the output voltage and current waveforms at PV fed to the HFI. In misalignment conditions, PV output voltage and efficiency system decreases. Figure 14b, c present the voltage and current waveforms at the ESS port and load side of the WCS port respectively. After misalignment condition the voltage and current waveforms.

By analyzing Figs. 13 and 14, it is evident that the misalignment effect on the PV system is minimal. Under misalignment, the SS-compensated WCS system delivers less power compared to the LCC-S compensation. At full load, SS compensation shows higher efficiency. The significant difference is seen at the ESS port, where the LCC-S compensation maintains higher power flow during misalignment, avoiding power losses. In contrast, with SS compensation, misalignment results in greater power drawn from the PV, which reduces the overall power available at the ESS port.

**Fig. 15:**
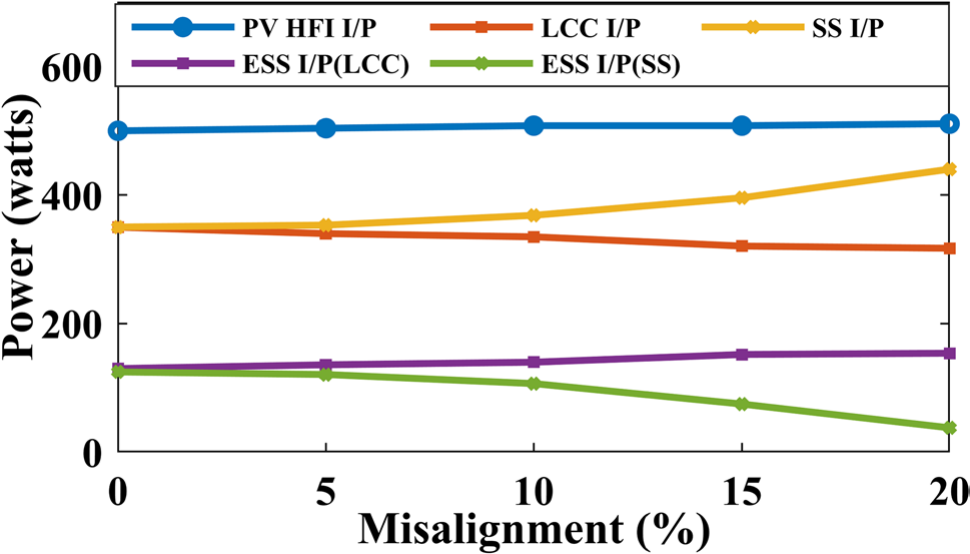
Power sweep at different misalignment conditions at only PV as source.

**Fig. 16:**
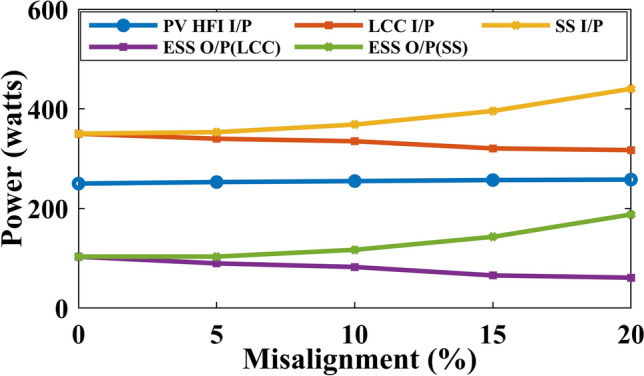
Power sweep at different misalignment conditions at PV and ESS as the source.

Figure 15 shows the power sweep at various misalignment conditions with the PV operating at full load. As observed in the figure, as misalignment increases, more power is drawn from the PV when using the proposed TPC with SS compensation. In contrast, with LCC-S compensation, as misalignment increases, the power drawn from the PV decreases, and the remaining power is directed to the ESS port. Figure 16 illustrates similar behavior when the PV operates at 50% load, with the ESS port supplying the remaining power. Under LCC-S compensation, the power drawn from the ESS port is minimal.

Table 4 presents a comparison of existing methods. While fully isolated TPCs exist, they are primarily designed for plug-in charging and other applications. Studies on TPC with wireless charging are negligible. Marei et al. proposed a TPC with wireless charging; however, their system is not fully isolated and does not effectively minimize circulating currents. As highlighted in the literature, most existing TPC methods using transformer-based plug-in charging systems have demonstrated maximum efficiencies of around 94%^26,27^. However, TPC systems with wireless charging and partially isolated configurations generally report lower efficiencies, typically around 83%^14^. In comparison, the proposed system achieves an improved efficiency of 88%, offering a well-balanced trade-off between electrical isolation and overall performance.

**Table 4:** No of turns of WPT pads.

References	Wireless integrated	Three-port transformer	Power	Advantages	Disadvantages
^14^	Yes	no	100 W	Bidirectional power transfer	No isolation between PV and ESS Circulating currents
^26^	No	Yes	2.3 kW	Bidirectional power transfer	No ESS system Circulating currents
^27^	No	Yes	300 W	Bidirectional ESS	Unidirectional Charger
Proposed	Yes	Yes	500 W	Bidirectional ESS	Unidirectional Charger

Based on the analysis, the PV-integrated TPC system with LCC-S compensation outperforms SS compensation in wireless charging systems under misalignment conditions, offering superior power flow management and higher efficiency. The misalignment effect on the PV system is minimal, but SS compensation draws more power from the PV, reducing the available power at the ESS port. In contrast, LCC-S compensation effectively channels more power to the ESS port, minimizing losses. Therefore, LCC-S compensation is recommended for optimal performance and efficiency in misaligned wireless charging applications. For scenarios without misalignment, series compensation can be considered a suitable alternative.

## Conclusion

In conclusion, this paper introduces a three-port series resonant converter (TPC) as an efficient interface for renewable energy sources, loads, and energy storage systems. The analysis and experimental results demonstrate effective control of power flow through phase-shifting of the square wave outputs from the active bridges. The proposed TPC topology incorporates auxiliary switches at the ESS and WCS ports to eliminate circulating current between the battery and PV ports, thereby optimizing power flow management. Moreover, the study reveals that PV-integrated TPC systems with LCC-S compensation outperform SS compensation under misalignment conditions. While SS compensation draws more power from the PV, reducing the energy available to the ESS port, LCC-S compensation channels more energy to the ESS port, reducing losses and improving overall efficiency.

These advanced converter designs, especially with LCC-S compensation, significantly enhance the efficiency and reliability of power management in PV systems, making them ideal for sustainable energy applications. Integrating wireless charging capabilities into these systems unlocks new opportunities, particularly for electric vehicles (EVs), providing seamless and efficient charging solutions. This integration not only improves solar energy utilization but also lays the groundwork for more adaptable and sustainable energy solutions in the future.

## Data Availability

The datasets used and/or analyzed during the current study are available from the corresponding author upon reasonable request.
